# Past summer upwelling events in the Gulf of Oman derived from a coral geochemical record

**DOI:** 10.1038/s41598-017-04865-5

**Published:** 2017-07-04

**Authors:** Takaaki K. Watanabe, Tsuyoshi Watanabe, Atsuko Yamazaki, Miriam Pfeiffer, Dieter Garbe-Schönberg, Michel R. Claereboudt

**Affiliations:** 1 Department of Natural History Sciences, Faculty of Science, Hokkaido University, Sapporo, 060-0810 Japan; 2 Atmosphere and Ocean Research Institaute, The University of Tokyo, Kashiwa, 277-5564 Japan; 3 RWTH Aachen University, Geological Institute, Wuellnerstrasse2, 52056 Aachen, Germany; 4 Institute of Geosciences, University of Kiel, Ludewig-Meyn Strasse 10, 24118 Kiel, Germany; 5 Department of Marine Science and Fisheries, College of Agricultural and Marine Sciences, Sultan Qaboos University, Box 34, Al-Khod, 123 Sultanate of Oman

## Abstract

We used a high-resolution oxygen isotope (δ^18^O_coral_), carbon isotope (δ^13^C_coral_) and Sr/Ca ratios measured in the skeleton of a reef-building coral, *Porites sp*., to reveal seasonal-scale upwelling events and their interannual variability in the Gulf of Oman. Our δ^13^C_coral_ record shows sharp negative excursions in the summer, which correlate with known upwelling events. Using δ^13^C_coral_ anomalies as a proxy for upwelling, we found 17 summer upwelling events occurred in the last 26 years. These anomalous negative excursions of δ^13^C_coral_ result from upwelled water depleted in ^13^C (dissolved inorganic carbon) and decreased water-column transparency. We reconstructed biweekly SSTs from coral Sr/Ca ratios and the oxygen isotopic composition of seawater (δ^18^O_SW_) by subtracting the reconstructed Sr/Ca-SST from δ^18^O_coral_. Significant δ^18^O_SW_ anomalies occur during major upwelling events. Our results suggest δ^13^C_coral_ anomalies can be used as a proxy for seasonal upwelling intensity in the Gulf of Oman, which, driven by the Indian/Arabian Summer Monsoon, is subject to interannual variability.

## Introduction

The Gulf of Oman is located on the northeastern coast of the Arabian Peninsula and both the Arabian Sea and the Gulf of Oman are located in arid environments. The climate is dominated by the seasonal reversal of the Indian/Arabian Monsoon, which in turn governs the surface wind field of the Indian Ocean north of 10°S. The intensity and direction of the monsoon winds vary seasonally. During the southwest (SW) Monsoon develops during the boreal summer (from June to mid-September) and is characterized by strong airflow across the Arabian Sea that feeds moisture and rainfall to the Indian subcontinent.

The Indian/Arabian Summer Monsoon causes coastal upwelling bringing cooler temperatures, nitrified and saline water to the sea surface along the southern coast of the Arabian Peninsula. Upwelled water flows northward and affects the oceanic stratification of the Gulf of Oman through gyres and eddy systems that sweep into the Oman Sea^1^. The Northern Arabian Sea is therefore one of the most productive areas in the world^2^. The SW Monsoon is also the major climatic factor affecting the near-shore environment and areas of coral growth in Oman during the summer months^3^.

The high nutrient content of this water induces phytoplankton blooms. Satellite-based ocean color measurements show the temporal and spatial variability of the surface chlorophyll-a distribution along the coast of the Southern Arabian Peninsula^4^. In the Gulf of Oman, upwelling does not necessarily occur every summer^5–7^. In addition, observational records that allow us to understand the dynamics of upwelling events in the Gulf of Oman are scarce. Satellite based sea surface temperature (SST) in the Gulf of Oman did not reflect low SST excursions in summer measured by CTDs. (Fig. S1). Long-term and *in situ* records of primary production, salinity and temperature are necessary in order to understand upwelling events^7^. In this study, we used paleo-climatic reconstructions from coral geochemical records to provide a history of summer monsoon-driven upwelling variability in the Gulf of Oman.

The geochemical proxies in coral skeletal carbonate provide a long-term history of environmental variation, with high time resolution (2 weeks to a month)^8, 9^. Coral skeletal oxygen isotopes (δ^18^O_coral_) reflect SST and oxygen isotopes in seawater (δ^18^O_SW_)^10, 11^. Coral skeletal Sr/Ca ratios are used as SST proxies^12^. Sea-surface salinity (SSS) is derived from δ^18^O_SW_, which is generated by subtracting the temperature component (obtained from coral Sr/Ca) from δ^18^O_coral_^12^. Stable isotopes of carbon in coral skeletons (δ^13^C_coral_) are influenced by kinetic isotopic fractionation, vital effects (photosynthesis and respiration) and the carbon isotopic composition of dissolved inorganic carbon in seawater (δ^13^C_DIC-SW_)^13–16^. Coral skeletons are precipitated in isotopic disequilibrium with ambient seawater as a result of kinetic and vital effects. The kinetic effect selectively depletes ^12^C and ^16^O in coral skeletons and is particularly important when coral growth rates are very low (<4 mm per year)^13, 14^. Photosynthetic activities of zooxanthellae affect δ^13^C_coral_ by changing the carbon isotopes in the internal dissolved inorganic carbon pool of the coral^17^_._ A 50% weakening of solar radiation induces a decrease of approximately 0.5‰_VPDB_ in δ^13^C_coral_^18^. The amount of solar radiation received by the coral varies depending on incoming solar radiation, cloud cover and water transparency^17, 19, 20^. Upwelling can reduce water transparency and change the sea-surface δ^13^C_DIC-SW_. Therefore, upwelling events should be registered by the coral via a decrease in δ^13^C_coral_. We used a coral record from the Gulf of Oman to reconstruct the timing and frequency of upwelling events using high-resolution records of Sr/Ca ratios, δ^18^O_SW_ and δ^13^C_coral_ based on a 26-year-old coral core.

## Results and Discussion

**Figure 1:**
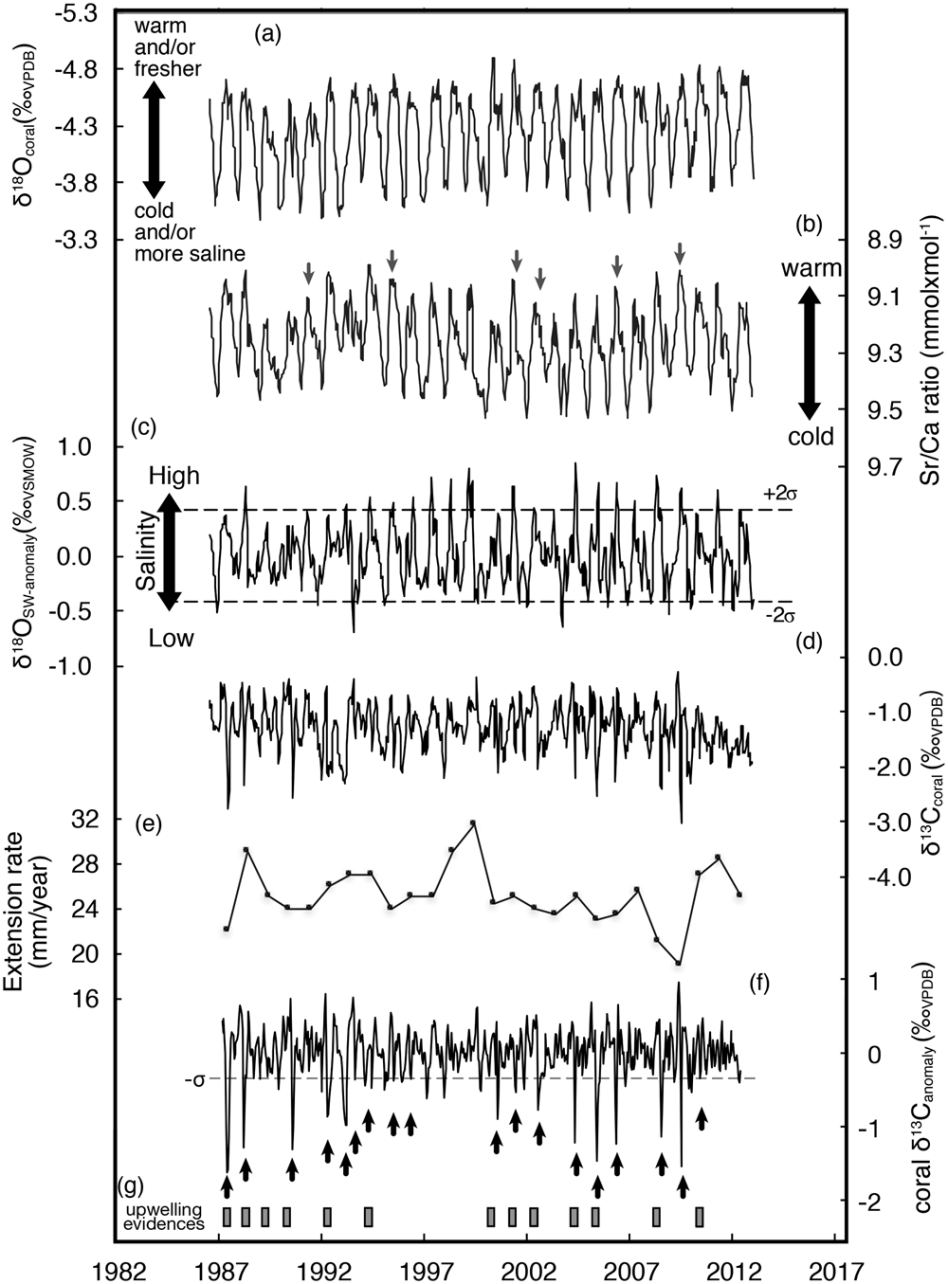
Oman coral proxy records and extension rate. (**a**) Coral skeletal δ^18^O_VPDB_ record, (**b**) Coral skeletal Sr/Ca ratio record. Grey arrows indicate the years of non-increasing Sr/Ca ratios in summer. (**c**) δ^18^O_SW-anomaly_, (**d**) Coral skeletal δ^13^C_VPDB_ record, (**e**) Extension rate calculated from distances between the anchor points in winter of each year. (**f**) Oman coral skeletal δ^13^C_VPDB_ anomaly. The timing of anomalous negative excursions of AN-δ^13^C in the summer are shown as black arrows. (**g**) *In situ* data showing low-SST and high chlorophyll-a (square symbols).

We determined δ^18^O_coral_, δ^13^C_coral_ and Sr/Ca ratios from 664 samples. Each powdered sample was split for paired stable isotope and Sr/Ca analysis. Sr/Ca ratios and δ^18^O_coral_ showed 26 distinct annual cycles (Fig. 1a and b). The average of the Sr/Ca ratios was 9.28 (mmol × mol^−1^), with values ranging from 8.98 to 9.56 (mmol × mol^−1^). The δ^18^O_coral_ averaged −4.33 (‰_VPDB_) and ranged from −4.92 to −3.41 (‰_VPDB_). We calculated the regression line between satellite SST and Oman coral Sr/Ca ratios using seasonal maxima and minima to avoid potential biases due to intra-seasonal age model uncertainties, as follows:

$$\mathrm{Sr}/\mathrm{Ca\; ratios}\,({\rm{mmol}}\times {{\rm{mol}}}^{-1})=-0.044\pm 0.003\,{\rm{SST}}+10.46\pm \mathrm{0.18.}({\rm{r}}=-0.95:{\rm{P}} < 0.01)$$

We established a regression line between satellite SST and δ^18^O_coral_, assuming that δ^18^O_coral_ reflect only SST variations, with the same δ^18^O_coral_ samples with Sr/Ca ratios, as follows:

$${\delta }^{18}{{\rm{O}}}_{{\rm{c}}{\rm{o}}{\rm{r}}{\rm{a}}{\rm{l}}}({\textperthousand }_{{\rm{V}}{\rm{P}}{\rm{D}}{\rm{B}}})=-0.104\pm 0.005\,{\rm{S}}{\rm{S}}{\rm{T}}-1.28\pm \mathrm{0.14.}\,({\rm{r}}=-0.92:{\rm{P}} < 0.01)$$

The correlation coefficient between δ^18^O_coral_ and Sr/Ca ratios was 0.77 (P < 0.01). δ^18^O_sw_ were calculated by subtracting the temperature component (estimated from coral Sr/Ca ratios) from δ^18^O_coral_, following the method proposed by Nurhati *et al*.^21^. The slope of the δ^18^O_coral_ -SST regression is −0.104 ± 0.005‰_VPDB_/°C, which is too high to be consistent with published estimates^12, 13, 22^. This suggests a significant contribution of δ^18^O_SW_ to δ^18^O_coral_. We therefore used the published regression slope of −0.18 ± 0.03 (‰/°C)^12^ to convert δ^18^O_coral_ to SST, and our slope of −0.044 mmol × mol^−1^/°C for SST estimation. The δ^18^O_SW_ anomalies were calculated by applying a band-pass filter to remove the periodicity components longer than 2 years and subtracting the seasonal cycle. Relative changes of δ^18^O_SW_ are on the order of ± 0.424‰_VSMOW_ (2σ). Anomalies above or below this threshold were marked as significant δ^18^O_SW_ anomalies (Fig. 1c). The uncertainty of calculated δ^18^O_SW_ is ± 0.113‰_VSMOW_ (following Nurhati *et al*.^21^).

The average δ^13^C_coral_ was −1.62 (‰_VPDB_) and ranged from −3.28 to +0.29 (‰_VPDB_). The δ^13^C_coral_ also showed clear seasonal variation (Fig. 1d) and distinct short-term negative anomalies (Fig. 1d). The δ^13^C_coral_ analysis was performed to avoid contamination from organic matter. We measured each CO_2_ gas sample 6 times using a dual inlet system loaded on a MAT251. Analytical precision of the δ^13^C_coral_ (standard deviations) were below 0.05‰. Growth rate disturbances and anomalous-colored annual band were not observed on X-ray photographs and coral cores. Therefore, the variations of δ^13^C_coral_ were assumed to reflect environmental changes rather than the coral growth disturbances^23^.

The interpretations of δ^13^C_coral_ has been debated about what the δ^13^C_coral_ values are reflecting^10, 13–16, 24, 25^. The main factors influence that can influence δ^13^C_coral_ include: (1) kinetic effect and vital effect, (2) solar radiation, (3) water-column transparency, (4) variation of δ^13^C_DIC-SW_ and (5) autotroph/heterotroph ratios.

Kinetic effects have been recognized as simultaneous ^18^O and ^13^C enrichment in coral skeletons with low extension rates^13^. Strong kinetic effects mask vital effects^13^. In our core, δ^13^C_coral_ values showed a weak negative correlation with the δ^18^O_coral_ record (r = −0.317, n = 634, P < 0.001: Fig. S2a). Summer δ^13^C_coral_ did not correlate significantly with δ^18^O_coral_ (r = 0.140, n = 181, P > 0.05: Fig. S2a). Winter δ^13^C_coral_ had no significant correlation with winter δ^18^O_coral_ (r = 0.04, P > 0.05, n = 159: Fig. S2b). The extension rates show that the Oman coral grew very quickly, on average 25.1 mm/year with a range between 19 to 31.5 mm. These values were considerably higher than the critical value estimated for kinetic isotopic fractionation effects (4 mm/year) (Fig. 1f)^14^. Therefore, the coral growth history and the lack of correlation between δ^13^C_coral_ and δ^18^O_coral_ suggest that the kinetic isotopic effect did not significantly affect this coral record.

Previous studies reported δ^13^C_coral_ on seasonal and inter-annual variations are attributable to solar radiation^17, 26^. To investigate the processes driving these δ^13^C_coral_ fluctuations, we compared δ^13^C_coral_ with satellite-based outgoing longwave radiation (OLR) (Fig. S3a) which reflect cloud cover. For a comparison of δ^13^C_coral_ with monthly-resolved OLR data, biweekly resolved δ^13^C_coral_ data were resampled at a monthly resolution using the software AnalySeries (version 2.0.8)^27^. The δ^13^C_coral_ were compared with OLR, and we calculated the correlation coefficients between these time series. δ^13^C_coral_ without anomalous δ^13^C_coral_ excursions positively correlated with OLR at a significant level (r = 0.411, P < 0.01, n = 302: Fig. S3a and S3b). A significant correlation appeared between the mean seasonal cycle of δ^13^C_coral_ and OLR averaged over the past 26 years (r = 0.702, P = 0.01, n = 12: Fig. S3c and d). The positive correlations between δ^13^C_coral_ and OLR (Fig. S2b and S2d) suggest that δ^13^C_coral_ captured the variation of photosynthetic activity caused by the seasonal solar radiation cycle. At inter-annual resolution, the 15 month-moving average profile of δ^13^C_coral_ positively correlate with that of OLR (r = 0.347, P < 0.01, n = 303: Fig. S4a and S4b). The duration of low OLR and coeval δ^13^C_coral_ decreased from 1992 to 1993. We propose that insolation and OLR had decreased in globally as a result of up-stirred volcanic aerosol from the eruption of Mount Pinatubo, the Philippines in June 1991^28^. Low δ^13^C_coral_ from 1992 to 1993 would be influenced by decreasing insolation which resulted from the volcanic eruption of Mount Pinatubo.

We calculated the δ^13^C_coral_ anomaly (δ^13^C_anomaly_) by removing the 15 month-moving average (31 bi-weekly data point) after subtracting the averaged seasonal cycle of δ^13^C_coral_. The threshold for δ^13^C_coral_ anomalous excursions was determined as a standard deviation of 1σ: ± 0.343‰_VPDB_. In summer, the anomalous negative excursions of the δ^13^C_anomaly_ occurred 17 times in summer, while 1 anomalous negative excursion occurred in the spring of 1993 (Fig. 1f). Anomalous positive δ^13^C_anomaly_ excursions were also observed prior to summer negative δ^13^C_anomaly_ excursions. The δ^13^C_anomaly_ had no significant correlation with OLR anomaly calculated by same procedure (r = 0.05, P > 0.3 Fig. S4c and S4d), suggesting that anomalous negative excursions of δ^13^C_anomaly_ in the summer (AN-δ^13^C) would not be generated from OLR variations.

We examined the timing of the AN-δ^13^C with the compiled evidence of each past upwelling event documented from *in situ* and satellite observations (Fig. 1f and g). Abrupt SST decreasing events in summer were revealed in 1987–1989^29^ and 2000^30^ from satellite SST data, in 1992^6^, 1994^4^, 2001^30^, 2002^30^ (Fig. S5) and 1990^31^ based on *in situ* SST data, and 2010 based on our vertical seawater temperature profile (Fig. S6). The vertical profile of seawater temperature deduced by temperature sensors attached to the diving gear of local volunteer divers in 2010, also suggest that the thermocline was closer to the surface during summer upwelling events (Fig. S6). In addition, Al-Azri *et al*.^1^ had measured chlorophyll-a concentrations, nutrients, phytoplankton density and SST in Fahal Island (23.67°N, 58.5°E) and Bandar Al Khayran (23.51°N, 58.72°E: near to our coral sample site). From July to September 2004, upwelling was observed as increasing chlorophyll-a concentrations and phytoplankton density as well as decreasing SST^1^. In August 2005, SST decreased for 1 month, while other parameters did not change^1^. Al-Azri *et al*., 2012 reported that *in situ* chlorophyll-a and satellite based SST suggested that upwelling also occurred in July, 2008^7^. The satellite observations (SeaWiFS and MODIS at 24°N, 58°E from Asia-Pacific Data Research Center^32^) from 1997 to 2013 suggested that chlorophyll-a concentrations in the Gulf of Oman increased in August 2000, September 2004 and August 2008 (Fig. S7). In other upwelling years, chlorophyll-a concentrations in satellite data were not available to compare with AN-δ^13^C due to the lack of satellite data in summer. Based on these *in situ* and satellite datasets, past upwelling events occurred in 1987^29^, 1988^29^, 1989^29^, 1990^31^, 1992^6^, 1994^6^, 2000–2002^30^, 2004^1^, 2008^7^ and 2010 (Fig. 1g). The AN-δ^13^C corresponds with these past upwelling events.

The possible controlling factors of the AN-δ^13^C with upwelling events are: (1) decreasing water-column transparency^33^, (2) variations of δ^13^C_DIC-SW_^16, 34^, and (3) change to heterotroph feeding^10^. It is known that increasing chlorophyll-a concentrations correspond with upwelling events^1, 7^ inducing phytoplankton blooms, thereby decreasing water-column transparency and depleting ^13^C_coral_ with low photosynthetic activities of zooxanthellae^33^. Moreover, lower δ^13^C_DIC-SW_ supply from greater depths decreases δ^13^C_DIC-SW_ at the sea surface^24, 25, 34^. Upwelling events may produce an AN-δ^13^C due to sudden decreases in water-column transparency and δ^13^C_DIC-SW_. Heterotrophic feeding would also be the controlling factor of negative δ^13^C _coral_ with upwelling events. A study^35^ reported that corals feeding ^13^C-depleted zooplankton decreased their δ^13^C_coral_. The coral records from the Gulf of Aqaba, Red Sea suggested that increasing heterotrophy with upwelling decreased δ^13^C_coral_ for an approximately half a year^10^. Afterwards, δ^13^C_coral_ could be increased by the preferential uptake of ^12^C by phytoplankton at the sea surface^16^. In the western Indonesian coast, it was reported that δ^13^C_coral_ increased by approximately 2.2‰_VPDB_ after large phytoplankton blooms due to upwelling^16^.

We propose the following mechanism to explain the short-term negative peaks in the δ^13^C_coral_: 1. Upwelling events bring deep, cold and nutrient-rich water with low δ^13^C_DIC-SW_ to the surface in summer. Upwelling events cause unusually high nutrient conditions in the Gulf of Oman. Photosynthesis activities in zooxanthella would be emphasized in eutrophic conditions and temporarily increased δ^13^C_coral_. 2. Lower δ^13^C_DIC-SW_ from the deep sea decreases δ^13^C_coral_. 3. Phytoplankton blooms arise from a nutrient supply to the sea surface. 4. Phytoplankton primarily depletes ^12^CO_2-SW_. Active phytoplankton photosynthesis increases ^13^CO_2-SW_. 5. δ^13^C_coral_ increases with the restoration of δ^13^C_DIC-SW_.

**Figure 2:**
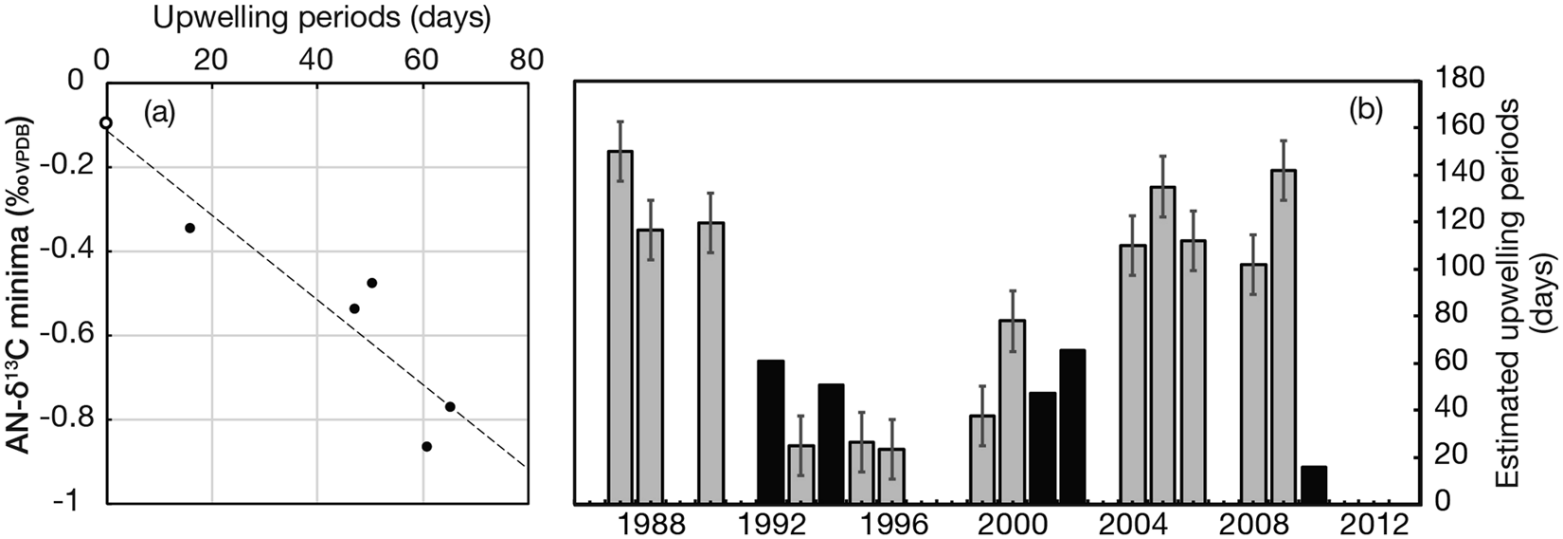
(**a**) Cross-plot of *in situ* low-SST periods and AN-δ^13^C minima. The dotted line indicates the regression between *in situ* low-SST periods and AN-δ^13^C minima (filled circles) and estimated δ^13^C_anomaly_ value for no upwelling periods (r = −0.937, P < 0.05, n = 6). The δ^13^C_anomaly_ value for no upwelling (open circle) was estimated from *in situ* δ^13^C_DIC-SW_ from the Arabian Sea^36^ and the value of δ^13^C in isotopic equilibrium between coral carbonate and seawater^37^ (**b**) Estimated upwelling periods from AN-δ^13^C minima. Black bars indicate the years which were used for the regression between *in situ* low-SST periods and δ^13^C_anomaly_ minima.

We compared the AN-δ^13^C minima with the upwelling periods (number of the days) in summer (Figs 2a, S5 and S6). *In situ* daily to weekly SST data in 1992, 1994, 2001, 2002 and 2010 revealed that SST during upwelling events was as same as winter SST (23.5 °C), and daily fluctuations of SST in upwelling periods ranged within 3 °C. Therefore, the numbers of the days for upwelling periods were defined as the duration of SST lower than 26.5 °C in summer. δ^13^C_anomaly_ values of no upwelling years (0 days) was estimated from *in situ* δ^13^C_DIC-SW_ in Arabian Sea (+1.325‰_VPDB_ at 0–10 m depth in non-upwelling seasons^36^) and the value of δ^13^C in isotopic equilibrium between coral carbonate and seawater^37^. The AN-δ^13^C minima were correlated to the upwelling periods as below.

Upwelling periods (days) = −87.16 ± 16.40 × AN-δ^13^C minima (‰_VPDB_) − 4.92 ± 9.45 (r = −0.937, P < 0.05; Fig. 2a).

Then, past upwelling periods in the year with no *in situ* SST data were reconstructed from each AN-δ^13^C using this equation (Fig. 2b). The estimated uncertainty for reconstructed upwelling-periods was 12.66 days (1σ) including the analytical precisions of δ^13^C_coral_, the intercept and the slope of this equation. In 1987, 2006, 2008, 2009, each upwelling period was extremely long, over 120 days (Fig. 2b). In those years, coral extension rates decreased to 23 mm/year (Fig. 1e). The long upwelling events would therefore have a negative effect on coral extension rate due to eutrophic conditions and decreased water-column transparency.

We compared the reconstructed upwelling events from AN-δ^13^C (Fig. 2b) with Sr/Ca ratios and δ^18^O_SW-anomaly_ (Fig. 1b and c). Sr/Ca ratios showed 1-month increasing (cooling) in summer except in 1994, 2001, 2002, 2006, and 2009, however, these did not correspond to reconstructed upwelling events. In non-AN-δ^13^C (upwelling) years (1989, 1991, 1997–1998, 2003, 2007, 2011–2012), the δ^18^O_SW-anomaly_ was low in summer. Upwelling events in the Gulf of Oman are driven by the SW Monsoon, which causes strong seasonal winds parallel to the coast of Southern Oman in the Arabian Sea, while the associated Ekman transport creates strong upwelling along the coastal margins, bringing cold, nutrient-rich water to the surface^1, 4^. This upwelled water has indirect impacts on corals and reef areas farther north through gyres and eddy systems that sweep into the Oman Sea^1, 4^. In addition, upwelling may be influenced by vertical seawater density, depending on SST and SSS^38^. The δ^18^O_SW-anomaly_ record suggested that deep seawater did not reach the sea surface as low-density water masses might form a cap on the sea surface in the Gulf of Oman.

Observations suggest that the primary productivity of the Gulf of Oman is subject to inter-annual variability^1^, but long-term observational records are lacking. Our new δ^13^C_coral_ record captured past upwelling events and their periods in the Gulf of Oman for 26 years. Thus, coral skeletal archives fill an important gap in the observational record and have great potential for increasing our understanding of the upwelling mechanisms in the Gulf of Oman. Moreover, it is possible to reconstruct past SST, SSS and upwelling frequency/intensity during the Holocene (from 0 to 10 ka) by applying the same methods to fossil corals from the Arabian Peninsula.

## Methods

### Coral sampling

**Figure 3:**
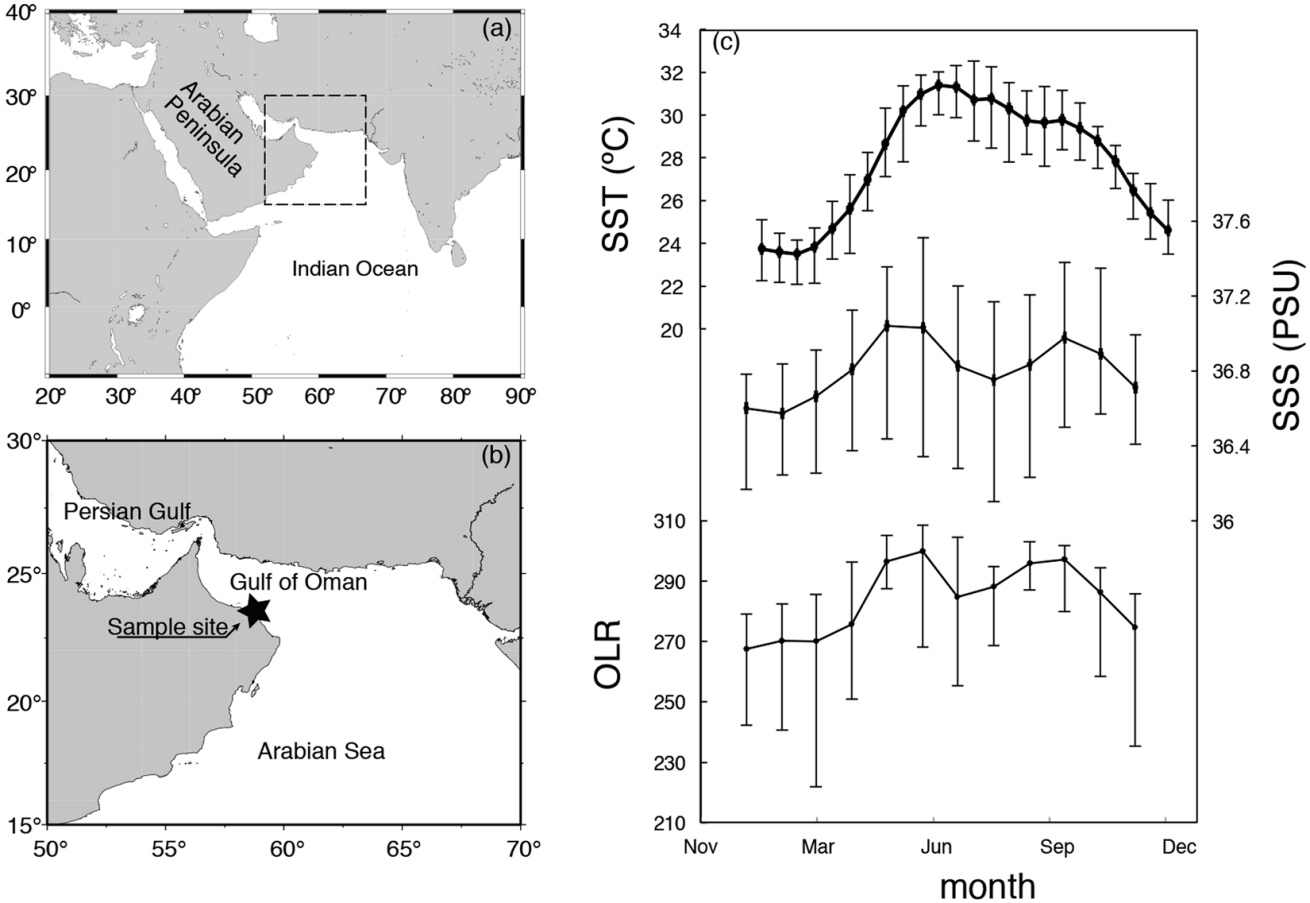
(**a**),(**b**) Map of the sampling site in the Gulf of Oman. The figures were generated using Generic Mapping Tools (GMT ver. 4.5.12)^44^. (**c**) Climatological data estimated from biweekly SST from 1987 to 2013 (data from AVHRR^39^), monthly SSS from 1987 to 2008 (data from SODA^40^) and OLR^41^ during past 26 years (data from https://climexp.knmi.nl/). Error bars indicate climatology deviation (1σ).

On February 23, 2013, we drilled a *Porites sp*. coral colony in the Gulf of Oman (23°30′ N, 58°45′ E: Fig. 3a and b). This *Porites* colony was living at a 2 m water depth in a small bay (Bandar Khayran) south of Muscat. There was no dry-riverbed (locally name: *wadi*) nearby; thus, we excluded the influence of occasional plumes of freshwater from coastal runoff at the site. In total, the coral core was 71 cm long. On the sampling date, we measured *in situ* SST and SSS at 24.3 °C and 38.2 PSU (practical salinity unit). Meteorological records from the weather station at Seeb Airport (23.60°N, 58.30°E) showed low precipitation rates, with less than 14.0 mm/month (the monthly average precipitation climatology for the past 23 years was 0.28–14.0 mm/month; GHCN-Monthly ver. 2). For coral proxy calibration, we used Advanced Very High Resolution Radiometer (AVHRR) satellite SST data, SODA satellite SSS data (http://iri.columbia.edu: Fig. 3c) and OLR data (https://climexp.knmi.nl/: Fig. 3c)^39–41^. Salinity records decrease in summer suggesting a possible occurrence of upwelling events.

### Subsampling

**Figure 4:**
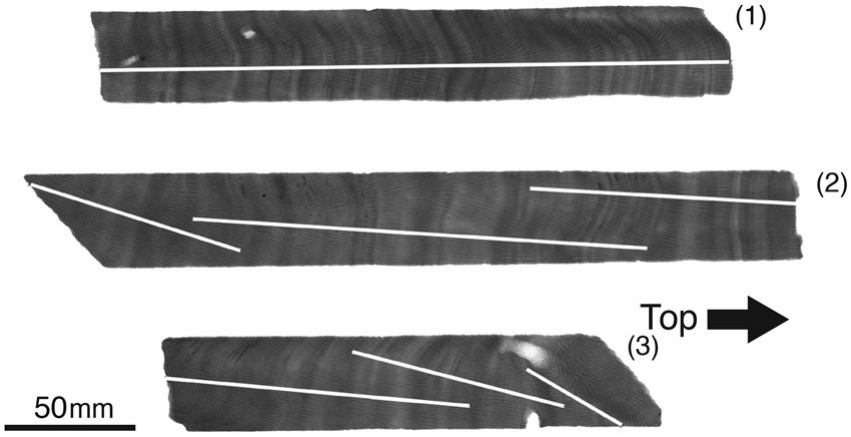
X-radiograph of coral core OMN130221 (*Porites sp*.). The white line indicates the measurement lines. Several overlapping measurement lines were sampled to ensuring reproducibility.

The coral core was sliced into 5-mm-thick slabs. We took X-radiographs of the coral slabs to identify the coral growth axis (Fig. 4). We prepared ledges of 1.5 mm in thickness along the maximum growth axis and obtained coral powder at a resolution of 0.5 mm for geochemical analysis.

### Oxygen and carbon isotope measurements

The coral powder was weighed, and 100 μg (±20 μg) were taken for oxygen and carbon stable isotope analysis. The sample powder was reacted with 100% H_3_PO_4_ at 70 °C in an automated carbonate preparation device (Kiel II). The δ^13^C_coral_ and δ^18^O_coral_ were analyzed with a Finnigan MAT251 stable isotope ratio mass spectrometer system installed at Hokkaido University. Analytical errors for δ^13^C_coral_ and δ^18^O_coral_ were determined to be 0.08 and 0.07‰, respectively, based on replicate measurements of the NBS-19 standard (1σ, n = 40).

### Trace element measurements

We measured Sr/Ca ratios with a SPECTRO CIROS CCD SOP inductively coupled plasma optical emission spectrophotometer installed at Kiel University following a combination of methods described by Schrag^42^ and de Villiers *et al*.^43^. Approximately 250 μg of coral powder was dissolved in 4 mL of HNO_3_. The sample solution for the measurement of trace elements was prepared via serial dilution with 2% HNO_3_ for a Ca concentration of ca. 8 ppm. Analytical precision of the Sr/Ca determinations was 0.07% RSD or 0.01 mmol × mol^−1^ (1σ).

### Data analysis

We used the coral Sr/Ca ratios to develop an age model for all proxies. Minima and maxima of the coral Sr/Ca ratios were chosen as anchor points and tied to the maxima and minima of SST, respectively. To obtain a time series with equidistant time steps, we resampled the proxy data at a biweekly resolution using the AnalySeries software, version 2.0.8^27^. Annual extension rates were estimated from the distance (in mm) between the winter anchor points in each sclerochronological year.

## Electronic supplementary material

Supplementary Information
