# Supplementary material for: Past summer upwelling events in the Gulf of Oman derived from a coral geochemical record
Source: Sci Rep. 2017 Jul 4;7:4568. doi: 10.1038/s41598-017-04865-5 (PMC5496871; doi:10.1038/s41598-017-04865-5)
Supplement: Supplementary file 1 — Supplementary Information [file 41598_2017_4865_MOESM1_ESM.pdf]

Supplementary Information for
Past summer upwelling events in the Gulf of Oman derived from a coral geochemical record

Takaaki K Watanabe[1], Tsuyoshi Watanabe[1†], Atsuko Yamazaki[1,2], Miriam Pfeiffer[3], Dieter Garbe-Schönberg[4] and Michel R Claereboudt[5]

[1] Department of Natural History Sciences, Faculty of Science, Hokkaido University, Sapporo 060-0810, Japan.

[2] Atmosphere and Ocean Research Institute, The University of Tokyo, Kashiwa 277-5564, Japan.

[3]RWTH Aachen University, Geological Institute, Wuellnerstrasse2, 52056 Aachen, Germany.

[4] Institute of Geosciences, University of Kiel, Ludewig-Meyn Strasse 10, 24118 Kiel, Germany.

[5] Department of Marine Science and Fisheries, College of Agricultural and Marine Sciences, Sultan Qaboos University, Box 34, Al-Khod, 123, Sultanate of Oman

Contents of this file: Figure S1, Figure S2, Figure S3, Figure S4, Figure S5, Figure S6 and Figure S7

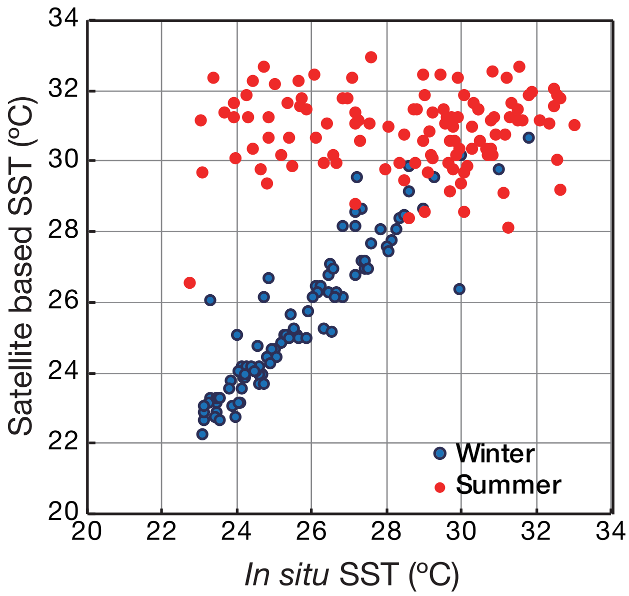

Figure S1. Scatterplot of SST measured by CTD vs. SST based on satellite in the Gulf of Oman during 10 years. Winter SST had a high correlation between *in situ* SST and satellite-based. Whereas for summer temperature, the correlation between *in situ* SST and satellite was nearly 0.

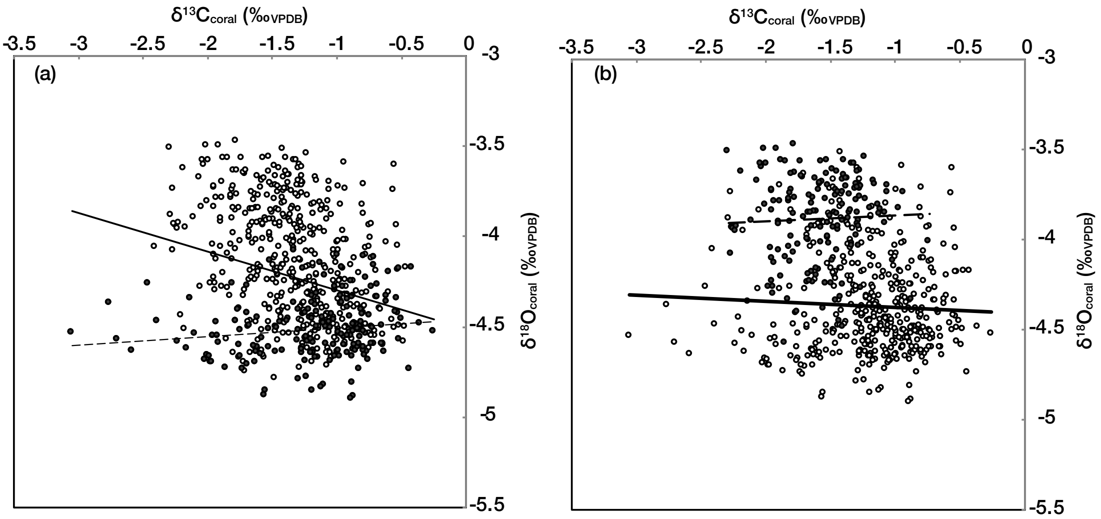

Figure S2. (a)Scatterplots of $\delta^{13}C_{coral}$ vs. $\delta^{18}O_{coral}$ with all data (white dots) and summer values only (black dots). A significant positive correlation is not observed in either scatterplot.

(b) Scatterplots of $\delta^{13}C_{coral}$ vs. $\delta^{18}O_{coral}$ without winter (open circle) and winter values (filled dots). Significant correlation was not confirmed.

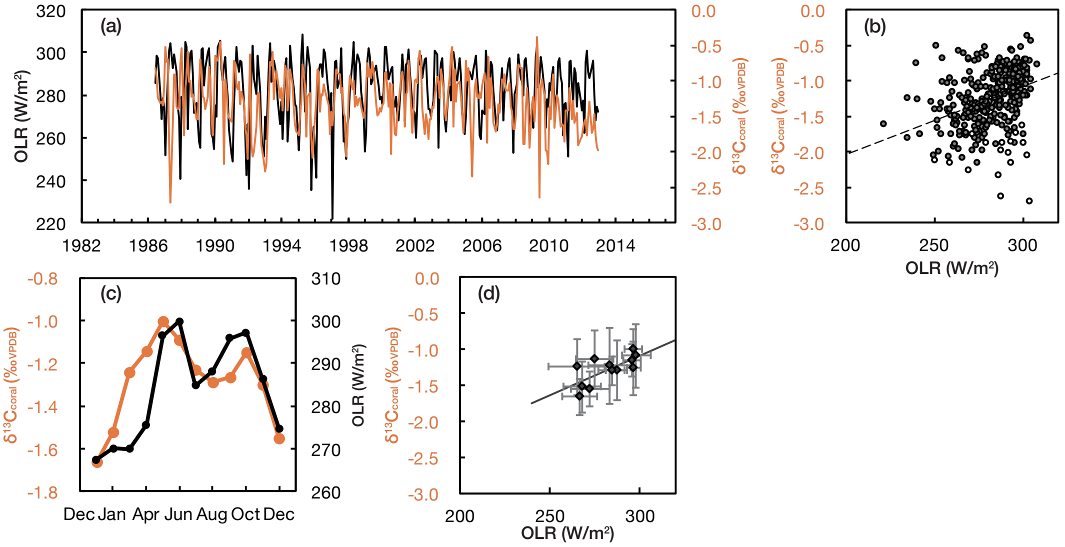

Figure S3. (a) OLR[41](orange line) and $\delta^{13}C_{coral}$ (black line). (b) Scatterplot of $\delta^{13}C_{coral}$ and OLR data[41]. Solid circles: $\delta^{13}C_{coral}$ vs. OLR for the monthly time series from 1986 to 2013 without anomalous $\delta^{13}C_{coral}$ peaks ($r = 0.411$, $P < 0.01$). (c) The climatologies of OLR (orange line) and $\delta^{13}C_{coral}$ (black line) from 1986 to 2013. (d) Monthly mean data of $\delta^{13}C_{coral}$ vs. OLR. The error bar indicates the standard deviation (1σ) of the monthly mean values ($r = 0.702$, $P < 0.01$) This averaged seasonal variations of $\delta^{13}C_{coral}$ and OLR were calculated from these dataset during 1986-2013. Regression lines were shown in each diagram.

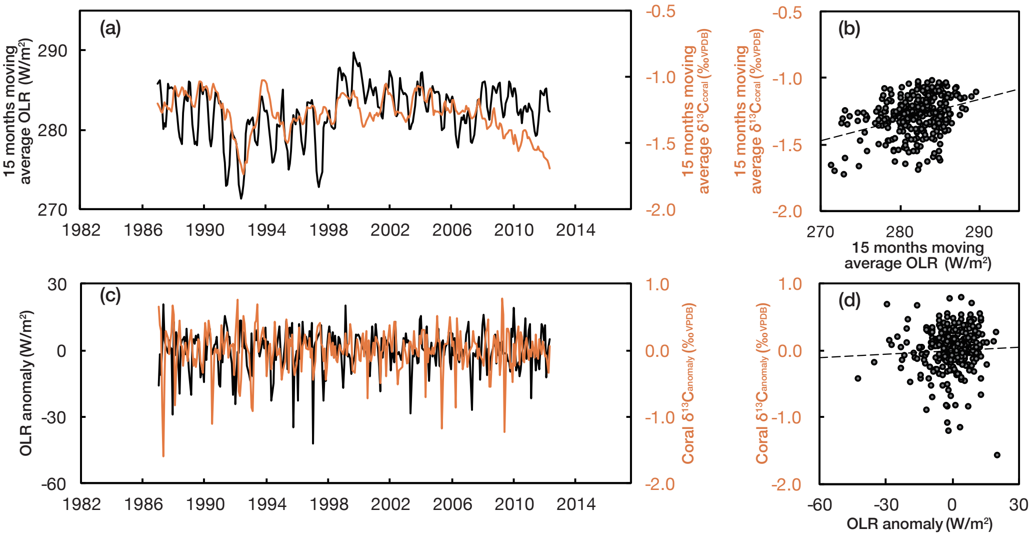

Figure S4. (a) The 15 months moving average profile of OLR[41] (orange line) and $\delta^{13}C_{coral}$ (black line). (b) The scatter diagrams between moving averaged OLR[41] and $\delta^{13}C_{coral}$. (c) $\delta^{13}C_{anomaly}$ (orange line) and OLR anomaly (black line). (d) Scatterplots of $\delta^{13}C_{anomaly}$ vs. OLR anomaly. Anomaly dataset showed no correlations ($r = 0.09$, $P > 0.05$).

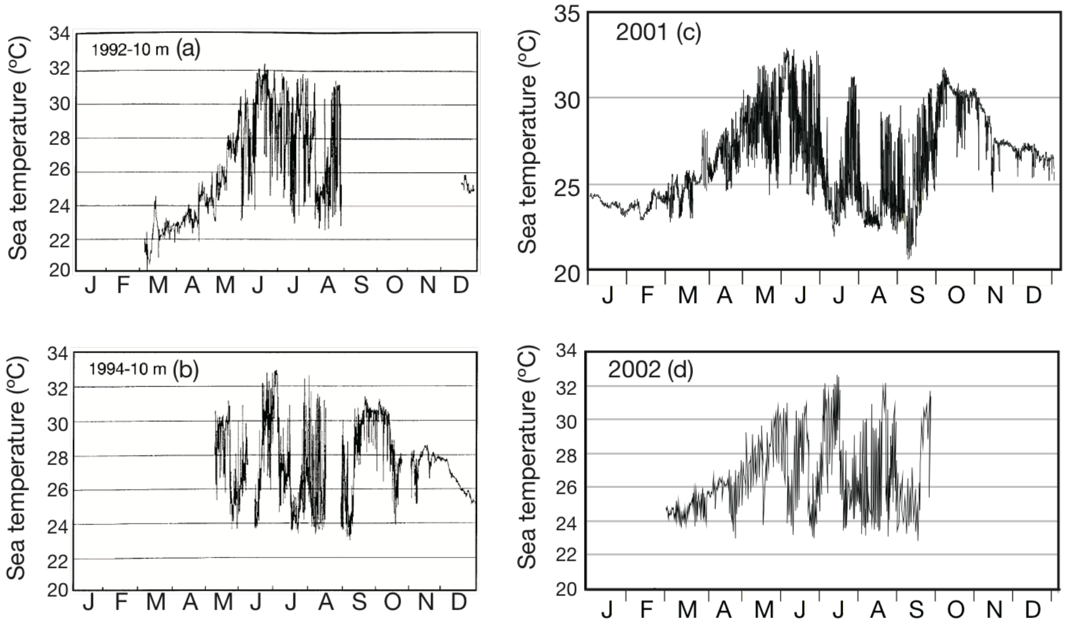

Figure S5. *In situ* seawater temperature profile at Fahal Island (23.67ºN, 58.5ºE) in 1992 at 10m depth (a) and 1994 at 10m depth (temperature data from Coles, 1997[6]) (b). *In situ* SST at Qalhat, Oman in 2001 at 6m depth (c) and 2002 at 6m depth (unpublished data, Claereboudt) (d). Seawater temperature profiles in 1992 ,1994 ,2001, and 2002 recorded upwelling-deduced abrupt decreases in summer.

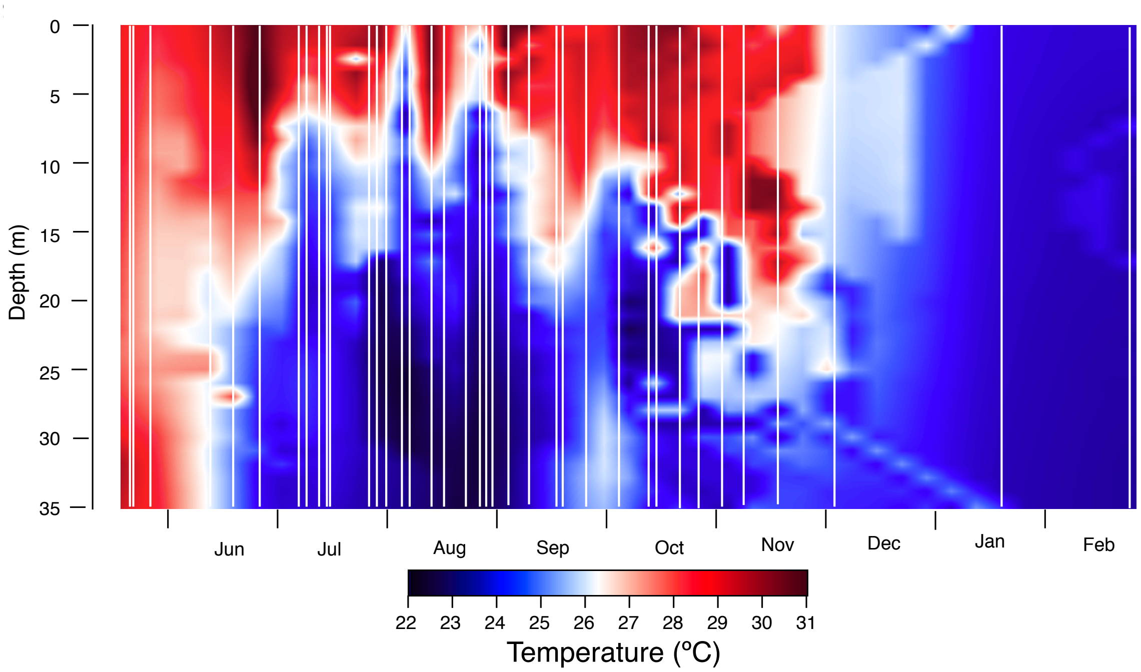

Figure S6. The vertical profiles of seawater temperature nearby coral sampling site in 2010. The depth profiles of temperatusing gear of local volunteer divers.

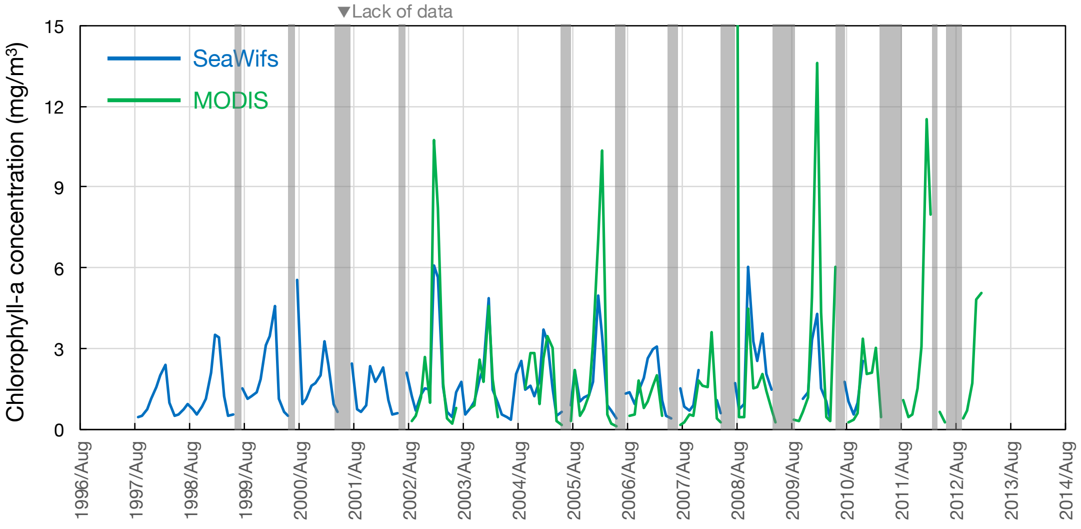

Figure S7. Chlorophyll-a concentrations from 1997 to 2013 based on satellite observations[32] (Blue line: SeaWiFS, Green line: MODIS at 24N, 58E). Grey bars indicated the periods of a lack of data.
